# The prevalence and impact of chronic neuropathic pain on daily and social life: A nationwide study in a Japanese population

**DOI:** 10.1002/ejp.977

**Published:** 2017-01-20

**Authors:** S. Inoue, T. Taguchi, T. Yamashita, M. Nakamura, T. Ushida

**Affiliations:** ^1^ Multidisciplinary Pain CentreAichi Medical UniversityJapan; ^2^ Department of Orthopaedic SurgeryYamaguchi UniversityJapan; ^3^ Department of Orthopaedic SurgerySapporo Medical University School of MedicineJapan; ^4^ Department of Orthopaedic SurgerySchool of MedicineKeio UniversityShinjukuTokyoJapan

## Abstract

**Background:**

This study marks the first epidemiological evaluation of the prevalence and burden of chronic neuropathic pain (NeP) in an Asian population. The objective of this nationwide cross‐sectional study was to identify the characteristics of individuals with NeP, detect the NeP features that affect their quality of life (QOL), and demonstrate the negative effects of NeP on social and daily living as well as comorbidities including depression, anxiety and sleep disorders.

**Methods:**

We mailed a cross‐sectional, population‐based epidemiological survey to a random nationwide sample of 10,000 Japanese adults over 20 years old.

**Results:**

The response rate was 54.4% (2445 men, 2992 women; mean age, 53.4 years). Prevalence of chronic pain was 16.6%, and prevalence of NeP was 3.2% as detected by the PainDETECT. Participants with NeP showed significantly lower quality of life according to scores on the EuroQol‐5 Dimensions scale (*p* < 0.001), higher levels of psychological distress on the Kessler 6‐item psychological distress scale (*p* < 0.001), poorer sleep quality (*p* < 0.001), and more workdays lost (*p* < 0.001) than did participants without NeP. Linear regression modelling showed that widespread pain, thermal hyperalgesia and pressure‐induced pain had strong associations with lower QOL, with regression coefficients of −0.046 (*p* < 0.001), −0.038 (*p* < 0.001), and −0.040 (*p* < 0.001), respectively.

**Conclusions:**

This study is the first to report the prevalence of NeP in an Asian population using a validated questionnaire. This study provides compelling evidence that chronic NeP is more strongly associated with poorer QOL, mental health and social well‐being than CP without a neuropathic component.

**Significance:**

This population‐based nationwide epidemiological study revealed the prevalence, characteristics, and negative effects of chronic pain with neuropathic components in Asian society. The prevalence of neuropathic pain was 3.2% with PainDETECT.

## 1 Introduction

Chronic neuropathic pain (NeP) can significantly reduce quality of life (QOL) and impose economic burdens on individuals and society. Strong evidence suggests that patients with NeP experience worse health‐related QOL than the general population (Jensen et al., 2007; Smith et al., 2007; Doth et al., 2010). Some population‐based epidemiological studies have also reported the negative effect that NeP has on health conditions. A nationwide mail‐in epidemiological survey of the general population in France showed that respondents with NeP also displayed lower QOL, more symptoms of anxiety and depression, and poorer sleep quality than those without NeP (Attal et al., 2011). The social background (e.g. family composition, job, education, ethnicity, and country of residence) of the respondents might affect their QOL.

Although the burden of NeP has been investigated in Western countries, the prevalence, severity, and negative effects of NeP remain unknown in Asian populations. Current estimates of the population prevalence of chronic NeP as a clinical entity have been performed in Europe (Smith et al., 2007; Bouhassira et al., 2008; Gustorff et al., 2008; Torrance et al., 2013), North America (Toth et al., 2009; Yawn et al., 2009) and Brazil (de Moraes Vieira et al., 2012). In Asia, Jih et al. (2009) conducted a cross‐sectional survey of herpes zoster in Taiwan and showed the incidence of post‐herpetic neuralgia, and Nakamura estimated that 20% of patients with chronic pain (CP) have NeP. Both studies investigated specific conditions and not general populations. Therefore, we conducted the first epidemiological study to estimate the prevalence and burden of NeP in a general Asian population using a validated questionnaire.

Neuropathic pain is a global burden. In a systematic review, van Hecke et al. (2014) found that the worldwide prevalence of chronic NeP varied extensively, ranging from 1.3% (Gajria et al., 2011) to 17.9% (Toth et al., 2009). Such variation is likely because of not only the disparate areas in which the investigations were conducted but also the different sociodemographic characteristics, methods of data collection, and definitions of CP. Most studies define CP as any persistent or intermittent pain that lasts >3 months, regardless of intensity or frequency. Thus, these estimates contain cases of mild and temporary pain as NeP. As a result, those estimates thus contain mild and temporary pain as cases of neuropathic pain. Breivik et al. (2006) conducted a large‐scale CP survey of 15 European countries and Israel and reported a prevalence of CP of 19%, in which CP was defined as a ‘pain severity of ≥5 on a scale of 1–10, with a duration of ≥6 months, featuring pain at least twice a week within the past month’. We applied Breivik's criteria regarding the chronicity of NeP to focus on more severe, long‐term pain.

The objective of this nationwide cross‐sectional study was to determine the prevalence of chronic NeP, identify the demographic characteristics of individuals who suffer from NeP, and demonstrate the negative effects that NeP has on the quality of the social and daily lives of a general Asian population.

A more complete understanding of refractory NeP and its effects on this population might lead to a greater awareness of its existence and effect, leading to future improvements in pain recognition and management.

## 2 Methods

We conducted a cross‐sectional population‐based postal survey of the Japanese population from 15 October 2013 to 5 November 2013. This nationwide epidemiological survey assessed a random sample of individuals across Japan. We used a mail‐in survey panel designed by Nippon Research Centre (Tokyo, Japan) that reflected the demographic composition of the Japanese population (http://www.nrc.co.jp/english/services/scheduled.html#panel). The panel consisted of a random address‐based sample; the sex and age distributions were similar to those of the national population. The questionnaire was mailed to 10,000 adults 20 years of age or older, with quotas set based on sex, age and regional distributions. Questionnaires were mailed from the Nippon Research Centre on behalf of the authors. No reminder letters were sent.

### 2.1 Questionnaire

The questionnaire used in this survey contained questions to determine information regarding the basic demographic characteristics of the participants (sex, age, location, occupation and so on) and information concerning their pain (severity, characteristics, location, duration, presence/absence of treatment, treating medical facility and presence of numbness at the pain site). Participants were asked about their pain intensity using an 11‐point numerical rating scale (NRS), where 0 = no pain and 10 = the worst pain imaginable.

Quality of life and distress scales were also used. Subjective health‐related QOL was assessed using the EuroQol‐5 Dimensions scale (EQ‐5D; The EuroQol Group 1990), which was developed in Europe. The EQ‐5D consists of five dimensions: mobility, self‐care, usual activities, pain/discomfort and anxiety/depression. Each dimension has three levels of response or severity (no problems, some problems or extreme problems). The combination of responses describes 243 different health states, with a set of values ranging from 1 (no problem in any dimension) to −0.111 (severe problems in all five dimensions), anchored by 0 (death) and 1 (full health), with a higher score indicating higher QOL, although scoring rules permit scores <0 for extremely impaired health states. The Japanese EuroQol Translation Team developed the Japanese version of the value set based on a survey of time trade‐off assessments for the population of Japan (Ikeda and Ikegami, 2001).

Participants rated the sleep interference caused by their pain over the past 30 days on the 11‐point NRS as a sleep interference score, where 0 = ‘pain does not interfere with sleep’ and 10 = ‘pain completely interferes with sleep’ (van Seventer et al., 2006).

The Kessler 6‐item (K6) psychological distress scale was also used (Shutty et al., 1992). The K6 includes items that measure the presence of nervousness, hopelessness, irritability, negative affect, fatigue and worthlessness experienced over the past 30 days. Items are rated on a 5‐point scale, where 0 indicates the absence of the symptom, and 4 indicates that the symptom was constant over the past 30 days. As a result, the final score on the K6 can range from 0 to 24, with higher scores indicating higher levels of psychological distress. A K6 score over 5 is considered a risk factor for a mood disorder in the Japanese population (Furukawa et al., 2008).

### 2.2 Definitions of CP and NeP

Participants who met the following three criteria were assigned to the CP group outlined by Breivik et al. (2006)): (1) pain lasting ≥6 months (excluding toothache, migraine, and menstrual pain); (2) pain experienced over the past month and at least twice over the past week and (3) pain intensity ≥5 on a 10‐point NRS during the most recent episode of pain. Participants with CP were divided into two groups according to NeP symptoms as assessed using the Japanese version of the painDETECT method (Matsubayashi et al., 2013) for the most troublesome pain. PainDETECT consists of nine self‐reported questionnaire items: seven weighted sensory descriptor items and two items related to the spatial and temporal characteristics of the individual's pain pattern. The sensitivity and specificity of the clinical diagnosis are 85% and 80%, respectively (Freynhagen et al., 2006). In a previous validation study of the Japanese painDETECT method (Takeshita et al., 2014), however, the sensitivity values associated with painDETECT scores of >19, >13, and >11 were 50%, 75% and 88%, respectively, in 122 patients with spinal cord injury. Participants were divided into three categories according to the following cut‐off points: score <13, non‐NeP (i.e. neuropathic component unlikely, <15%); scores ≥13 but <19, suspected NeP (i.e. possible involvement of NeP); score ≥19, NeP (i.e. neuropathic component likely, >90%). Thus, we divided CP into two subgroups: Participants with scores ≥13 were placed in the neuropathic components (NeP) group, and those with scores <13 were placed in the group without NeP, similar to prior studies (Hochman et al., 2013; Nakamura et al., 2014; Koop et al., 2015).

### 2.3 Statistical analyses

The data were analysed using SPSS version 21.0 for Windows (IBM, Armonk, NY, USA). Continuous data are reported as the mean ± standard deviation (SD) or standard error (SE). Analyses of variance and independent‐samples *t*‐tests were used to examine normally distributed variables. Categorical data are presented as *n* (%) and were analysed using Pearson's chi‐square test. A simultaneous logistic regression was performed to evaluate the effects of specific demographic characteristics, social factors and disease variables on pain status. This analysis produced odds ratios and 95% confidence intervals (CIs). Values of *p* < 0.05 were considered significant in all analyses. An analysis of covariance (ANCOVA), followed by Bonferroni *post hoc* tests, was conducted to determine the differences in health utilities among the three defined pain subgroups (no CP vs. CP without NeP; no CP vs. CP with NeP; and CP without NeP vs. CP with NeP) after adjusting for age, sex, intensity of pain, and duration of pain.

## 3 Results

### 3.1 Respondents

Surveys were completed and returned by 5553 individuals (response rate, 56.8%). We confirmed all data in detail and strictly excluded responses with missing data, reducing the final sample size to 5437 (overall response rate, 54.4%). No significant differences were observed between responders and non‐responders in terms of age, sex or residential area. The respondents were composed of 2445 men and 2992 women, with a mean age of 53.4 years old (range, 18–89 years old).

### 3.2 Rates of CP and NeP

Of the 5437 respondents, 3344 reported pain lasting >6 months (61.5%). The percentage of respondents with CP, as defined in the Methods above, was 16.6% (95% CI, 15.7–17.7%). According to the painDETECT scores, 173 respondents met the criteria for NeP (painDETECT ≥13); thus, the incidence was 3.2% (95% CI, 2.7–3.7%) among all respondents. The detailed percentages of the painDETECT scores in the NeP groups were as follows: score >18 (likely NeP), 0.7% (95% CI, 0.5–1.0%) and score 13–18 (possible NeP), 2.5% (95% CI, 2.1–2.9%).

### 3.3 Sociodemographic characteristics of the participants with NeP

**Figure 1:**
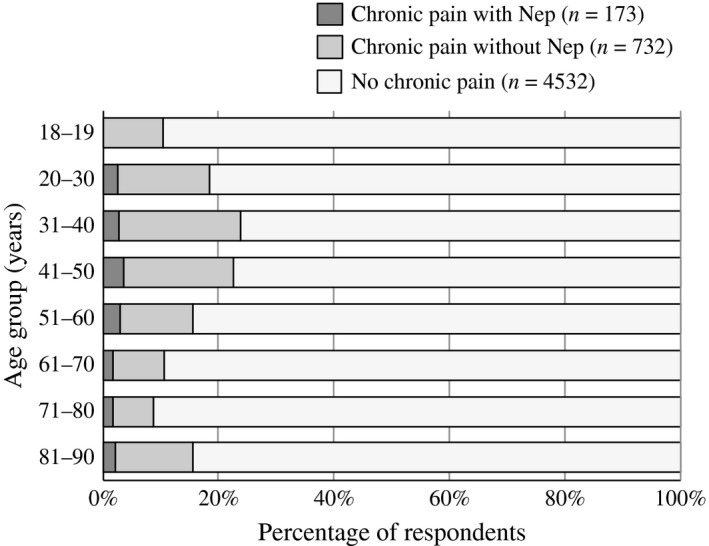
Percentage of respondents with chronic pain with or without neuropathic pain by age.

The background characteristics of the sample were determined for three subgroups: non‐CP, CP with NeP (painDETECT ≥13), and CP without NeP (painDETECT <13; Table 1). CP with or without NeP was more common in women (58.4% and 59.4%, respectively) than in men (41.6% and 40.6%, respectively). The mean age of the group with NeP was significantly higher (51.4 ± 15.7 years) than that of the group without NeP (48.0 ± 15.5 years, *p* < 0.001; Table 1). The frequency of NeP was highest among patients in their 40s and 50s (Fig. 1). CP with NeP did not significantly differ from CP without NeP on any of the measured sociodemographic characteristics (e.g. family composition, education, occupation or income).

**Table 1:** Sociodemographic characteristics of the respondents without CP (non‐CP), CP with NeP and CP without NeP

	All participants (*n* = 5437)	Non‐CP (*n* = 4532)	CP		With NeP vs. Without Nep
			With neuropathic pain components (*n* = 173)	Without neuropathic pain components (*n* = 732)	
	*n*	*n* (%)	*n* (%)	*n* (%)	*p*‐value
Sex					
Male	2445	2076 (45.8%)	72 (41.6%)	297 (40.6%)	0.869^a^
Female	2992	2456 (54.2%)	101 (58.4%)	435 (59.4%)	
Age (years, mean ± SD)	53.4 ± 16.8	54.2 ± 16.8	51.4 ± 15.7	48.0 ± 15.5	0.010^c^
Family composition					0.087^a^
Living alone	412	362 (8.0%)	11 (6.4%)	39 (5.4%)	0.590^b^
Living with spouse	1376	1213 (26.9%)	35 (20.4%)	128 (17.6%)	0.392^b^
Living with spouse and children	2974	2415 (53.5%)	93 (54.1%)	466 (63.9%)	0.017^b^
Living with others	652	523 (11.6%)	33 (19.2%)	96 (13.2%)	0.043^b^
Education					0.137^a^
Less than high school	435	361 (8.1%)	16 (9.4%)	58 (8.0%)	0.534^b^
High school	2464	2071 (46.2%)	84 (49.4%)	309 (42.4%)	0.096^b^
Collage/equivalent	2487	2055 (45.8%)	70 (41.2%)	362 (49.7%)	0.046^b^
Occupation					0.342^a^
Full‐time	2313	1895 (42.0%)	76 (43.9%)	342 (46.8%)	0.498^b^
Part‐time/House wife	2130	1772 (39.3%)	65 (37.6%)	293 (40.1%)	0.544^b^
Student	146	129 (2.9%)	4 (2.3%)	13 (1.8%)	0.642^b^
Unemployed	823	712 (15.8%)	28 (16.2%)	83 (11.4%)	0.082^b^
Income					0.007^a^
Low (0–$30,000)	1503	1278 (29.0%)	58 (34.7%)	167 (23.0%)	0.002^b^
Average ($30,001–$80,000)	3077	2545 (57.7%)	87 (52.1%)	445 (61.4%)	0.027^b^
High ($80,001–)	720	585 (13.3%)	22 (13.2%)	113 (15.6%)	0.433^b^
Regular alcohol consumption					0.645^a^
Current drinker	2617	2174 (48.3%)	78 (46.4%)	365 (50.1%)	0.395^b^
Ex‐drinker	550	441 (9.8%)	23 (13.7%)	86 (11.8%)	0.498^b^
Never drink	2228	1883 (41.9%)	67 (39.9%)	278 (38.1%)	0.675^b^
Smoking	5437				0.373^a^
Current smoker	930	730 (16.2%)	39 (22.9%)	161 (22.1%)	0.809^b^
Ex‐smoker	1009	834 (18.6%)	39 (22.9%)	136 (18.7%)	0.204^b^
Never smoked	3457	2933 (65.2%)	92 (54.1%)	432 (59.3%)	0.221^b^

SD, Standard Deviation.

*p*‐value: a, Pearson's Chi‐squared test; b, Residual analysis; c, Unpaired *t*‐test.

### 3.4 Pain characteristics

Pain intensity was significantly higher in participants who had CP with NeP (7.1 ± 1.2) than in those without NeP (6.1 ± 1.1), indicating that the former condition was more severe (*p* < 0.001) than the latter (Table 2). Although CP with NeP was associated with greater pain intensity than CP without NeP, we did not find a difference in pain duration between the two groups. The most common locations of pain (only one answer was allowed) in the CP with NeP group were the lower back and buttocks (35.3%), followed by the lower extremities (19.1%), neck (18.5%) and shoulders (16.2%). The frequencies of seven pain descriptors in the painDETECT (i.e. burning pain, prickling, mechanical allodynia, pain attacks, thermal hyperalgesia, numbness and pressure‐induced pain) were calculated (score >3, ‘strongly’ or ‘very strongly’). Numbness (28.3%) and prickling (27.8%) were the most frequent perceptions among the seven items. Respondents with NeP showed higher percentages in all sensory disturbances than those without NeP, and a significant difference was observed between the groups with regard to widespread pain (with NeP, 59.5%; without NeP, 24.6%). Linear regression modelling showed that widespread pain, thermal hyperalgesia, and pressure‐induced pain had strong associations with lower QOL, with regression coefficients of −0.046 (*p* < 0.001), −0.038 (*p* < 0.001), and −0.040 (*p* < 0.001), respectively (Table 3). In the case of participants with CP with NeP, EQ‐5D most affected burning pain (−0.019) and pressure pain (−0.010); numbness was not associated with this value (0.006).

**Table 2:** The characteristics of chronic pain with or without neuropathic pain

	Chronic pain		*p*‐value
	With neuropathic pain components (*n* = 173)	Without neuropathic pain components (*n* = 732)	
Pain intensity (VAS0‐10)	7.1 ± 1.2	6.1 ± 1.1	<0.001^a^
Pain duration (Months)	125.1 ± 119.5	111.7 ± 110.9	0.169^a^
Surgical history for pain considered most severe			0.011^b^
Yes	13 (7.6%)	22 (3.1%)	
No	158 (92.4%)	699 (97.0%)	
Location of pain considered most severe			0.245^b^
Head & face	5 (2.9%)	17 (2.3%)	0.654^c^
Neck	32 (18.6%)	181 (24.7%)	0.089^c^
Shoulders	28 (16.3%)	126 (17.2%)	0.769^c^
Chest & Abdomen	1 (0.6%)	6 (0.8%)	0.748^c^
Upper extremity	12 (7.0%)	26 (3.6%)	0.044^c^
Lower back and buttock	61 (35.5%)	265 (36.2%)	0.856^c^
Lower extremity	33 (19.2%)	111 (15.2%)	0.195^c^
PDQ pain descriptors (score >3, strongly or very strongly)			
Burning	16 (9.3%)	6 (0.8%)	<0.001^b^
Prickling	48 (27.8%)	17 (2.3%)	<0.001^b^
Mechanical allodynia	17 (9.8%)	1 (0.1%)	<0.001^b^
Pain attacks	41 (23.7%)	30 (4.1%)	<0.001^b^
Thermal hyperalgesia	8 (4.6%)	2 (0.3%)	<0.001^b^
Numbness	49 (28.3%)	45 (6.2%)	<0.001^b^
Pressure‐induced pain	35 (20.2%)	19 (2.6%)	<0.001^b^
Radiating pain	103 (59.5%)	180 (24.6%)	<0.001^b^

*p*‐value: a, Unpaired *t*‐test; b, Pearson's Chi‐squared test; c, residual analysis.

**Table 3:** Linear regression modelling seeking to identify the PDQ pain descriptors associated with the EQ‐5D

	B	SE	β	*p*
Overall (*n* = 5437)				
PDQ pain descriptors				
Burning	−0.033	0.002	−0.225	<0.001
Prickling	−0.022	0.002	−0.183	<0.001
Mechanical allodynia	−0.024	0.002	−0.149	<0.001
Pain attacks	−0.016	0.002	−0.144	<0.001
Thermal hyperalgesia	−0.038	0.003	−0.190	<0.001
Numbness	−0.020	0.002	−0.170	<0.001
Pressure‐induced pain	−0.040	0.002	−0.276	<0.001
Widely spread pain	−0.046	0.003	−0.232	<0.001
CP with neuropathic pain components (*n* = 173)				
PDQ pain descriptors				
Burning	−0.019	0.007	−0.213	<0.005
Prickling	−0.008	0.007	−0.091	0.232
Mechanical allodynia	−0.009	0.007	−0.098	0.201
Pain attacks	−0.006	0.007	−0.069	0.365
Thermal hyperalgesia	−0.014	0.008	−0.141	0.065
Numbness	0.006	0.008	0.056	0.465
Pressure‐induced pain	−0.010	0.005	−0.150	<0.005
Widespread pain	−0.005	0.009	−0.041	0.596

B, regression coefficient; SE, standard error; β, standardized regression, coefficients; *p*,* p*‐value.

### 3.5 Health‐related QOL (EQ‐5D, K6 and sleep interference score)

**Figure 2:**
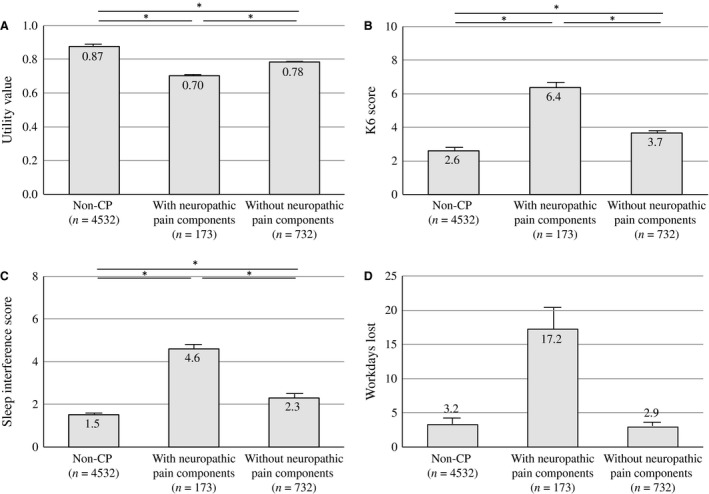
The influence of neuropathic pain components on the utility values of EQ‐5D (A), K6 (B), sleep interference (C) and workdays lost (D). Statistical analyses were performed with an ANCOVA followed by Bonferroni *post hoc* tests. Means are shown as columns, and the error bars represent the SE. *p < 0.001.

The effects of CP with and without NeP on QOL, psychological status, sleep quality and working situation are shown in Fig. 2. All respondents, regardless of whether they experienced CP, completed the EQ‐5D and K6 questionnaires. Regarding overall function, pain, and well‐being, participants with NeP reported a mean (±SD) score of 0.70 ± 0.11 on the utility index of the EQ‐5D. This score was significantly lower than that reported by those without NeP (0.78 ± 0.13) and non‐CP (0.87 ± 0.15), indicating that CP with NeP is associated with the poorest health status (Fig. 2A). In addition, the EQ‐5D scores of those with PainDETECT scores >18 (i.e. likely NeP) decreased to 0.65 ± 0.13. Respondents with CP with NeP reported significantly higher scores (6.4 ± 5.1) on the K6 than those with CP without NeP (3.7 ± 4.1, *p* < 0.001) or non‐CP (2.6 ± 3.4, *p* < 0.001). Those with CP with NeP showed the highest levels of psychological distress and presented with significant anxiety as defined as a score >5 (Fig. 2B). The sleep interference score for respondents with CP with NeP (4.6 ± 2.7) was higher than that for those with CP without NeP (2.3 ± 2.4, *p* < 0.001) or non‐CP (1.5 ± 2.2, *p* < 0.001; Fig. 2C). The mean number of missed workdays over the previous year due to pain in participants with CP with NeP was 17.2 ± 51.6 days, which was higher than that in participants with CP without NeP (2.9 ± 15.9, *p* < 0.001) or non‐CP (3.2 ± 22.6, *p* < 0.001; Fig. 2D).

### 3.6 Use of healthcare resources

**Figure 3:**
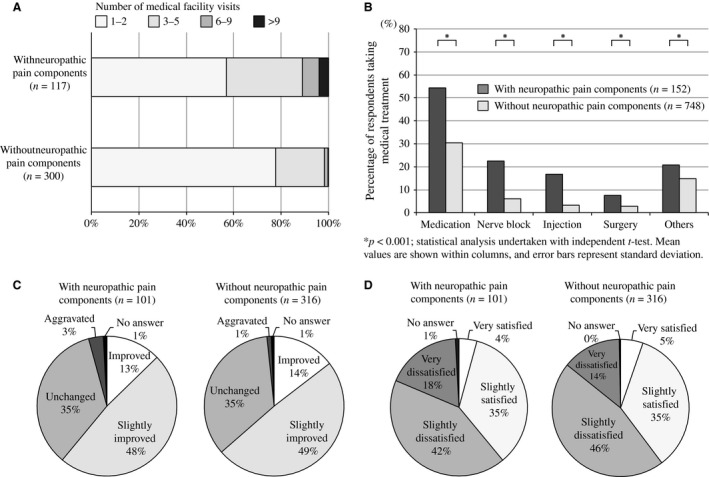
(A) Number of medical facility visits for the treatment of chronic pain. (B) Variety of medical treatments received for chronic pain (multiple answers allowed). (C) Number of medical treatment facility visits for chronic pain (CP). (D) Efficacy of treatment for CP.

Respondents with NeP tended to have more physician visits for their pain than those without NeP (Fig. 3A). The percentage of participants who visited more than three medical facilities for individuals who had CP with NeP (42.7%) was twice that for those who had CP without NeP (21.3%). Surprisingly, 3.4% of respondents with CP with NeP reported visiting more than 10 medical facilities. Higher proportions of respondents who had CP with NeP reported receiving pharmacotherapy, nerve block, intravenous injection and surgery compared with respondents without NeP (54.3% vs. 30.5%, 22.5% vs. 6.1%, 16.8% vs. 3.3%, and 7.5% vs. 2.9%, respectively; Fig. 3B). Despite the common use of medical resources among respondents with CP with NeP, the outcomes following medical treatment were similar to those without NeP. Conversely, the population of participants who reported the aggravation of pain after treatment was greater in respondents with NeP (Fig. 3C). The rates of satisfaction with medical treatment were approximately the same in the two groups, although the proportion who answered ‘very dissatisfied’ was higher among respondents with CP with NeP (Fig. 3D).

## 4 Discussion

### 4.1 Negative effects of chronic NeP on QOL

This study provides compelling evidence that the presence of a neuropathic component to CP is strongly associated with worse QOL, mental health and social well‐being. Outcomes collected directly from patients without interpretation by a clinician or another caregiver are known as ‘patient‐reported outcomes’ and are in widespread use in clinical research (Deshpande et al., 2011). Although medical technology allows for the measurement of physical, physiological and biochemical data from patients, these data cannot adequately represent patient suffering (McKenna, 2011). Furthermore, pain is a difficult outcome to measure because of its multifaceted and subjective nature (Mannion et al., 2007). Therefore, patient‐reported outcomes such as symptoms, health‐related QOL, or satisfaction with medical care can provide information about the effect of pain on the individual.

**Figure 4:**
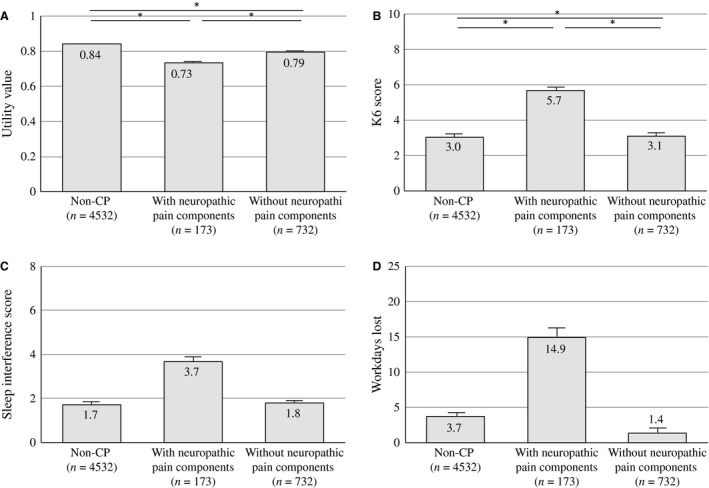
The influence of neuropathic pain (NeP) components on health‐related QOL, psychological distress, sleep and loss of work. The adjusted influence of NeP components on the utility values of EQ‐5D (A), K6 (B), sleep interference (C) and workdays lost (D). Values were adjusted by age, sex, intensity of pain and duration of pain. Statistical analyses were performed with an ANCOVA followed by Bonferroni *post hoc* tests. Means are shown as columns, and the error bars represent the SE. *p < 0.001.

In this study, respondents with CP with NeP experienced worse QOL, higher psychological distress, greater interference with sleep, and the loss of more workdays than those without NeP. Doth et al. (2010) reviewed 24 studies that investigated the health‐related QOL of patients with NeP using the EQ‐5D and reported an average health utility of 0.42 in a mixed population of patients with NeP, compared with a population‐based sample of 0.87 (Luo et al., 2005). In this study, participants with NeP reported a significantly lower utility index (0.70 ± 0.11) on the EQ‐5D than those without NeP (0.78 ± 0.13) and non‐CP (0.87 ± 0.15) in the general population. However, pain intensity was also higher in participants with NeP than those without NeP. McDermott et al. (2006) reported that pain severity is associated with worse EQ‐5D scores (NRS 1–3, 0.67; NRS 4–6, 0.46; and NRS 7–10, 0.16) in patients with NeP. Considering the McDermott report, the negative effects of chronic NeP should be compared between patients with and without NeP, after adjusting for age, sex, pain intensity and duration (Fig. 4A–D). As a result, our study indicated that the detrimental effects of chronic pain on a respondent's QOL are influenced not only by the intensity and duration of pain but also by the neuropathic nature of the pain.

The ultimate goal of treating CP is not to terminate the pain but to restore normal function, reduce comorbidities such as depression, anxiety and sleep disorders, and achieve better QOL. To achieve this goal, healthcare providers must pay close attention to both suffering and pain. Therefore, we must be more conscious of the type of pain in clinical pain treatment and provide different approaches and support based on the origin of pain.

### 4.2 The prevalence of chronic NeP

Although several epidemiological studies have been performed in Europe, this study is the first to report the prevalence of NeP in a general Asian population using a validated NeP questionnaire. This study showed that the prevalence of chronic NeP in a general Japanese population is 3.2%, which is lower than the 8.8% found in the United States (Yawn et al., 2009) and the 8.2% found in the United Kingdom (Torrance et al., 2006). However, those studies also reported a relatively high prevalence of CP with a duration of >3 months, representing 64% and 48% of the study samples, respectively. Those estimates included mild and temporary pain as cases of NeP because of the lack of criteria for pain severity in intensity and frequency. Bouhassira et al. (2008) provided two distinct prevalence rates in France: that of moderate‐to‐severe CP (31%) and of at least moderate pain (20%). Similarly, the prevalence of moderate‐to‐severe NeP is 6.9%, but for at least moderate NeP is only 5.1%, the latter more closely resembling the prevalence obtained by this study (3.2%) among participants with higher levels of pain (≥5/10). One of our goals was to determine the percentage of individuals who ‘suffer’ from NeP, not just those who have NeP. The overall prevalence of CP in this study was 16.6%, which is similar to that in Germany, the country with the median CP prevalence according to Breivik et al. who used the criteria for CP that reflected a population with long‐lasting serious pain. In other words, the population suffering from chronic ‘severe’ NeP in Europe can be estimated based on the rate of the current nationwide population survey. From this result, over 1 million people (1,024,000) in Japan had CP with NeP in 2013. This estimate was based on participants who met all the criteria for severe chronic NeP and suggests that more people have moderate or mild NeP. Accurate fundamental data regarding the prevalence and characteristics of NeP from a nationwide survey in accordance with each situation will allow us to formulate effective control measures from the perspectives of clinical medicine, public health and administrative policy.

### 4.3 The effective use of healthcare resources

Neuropathic pain is widely recognized as one of the most intractable pain conditions to manage. NeP results in significant disability, and clinical outcomes are often unsatisfactory (Finnerup et al., 2005; Dworkin et al., 2007; Attal et al., 2010; Nakamura et al., 2014). Despite spending more on healthcare resources, this study showed that participants with NeP did not experience better outcomes or satisfaction than those without NeP. Similarly, Schaefer et al. (2014) reported that participants with greater NeP severity use more healthcare resources in terms of both direct and indirect costs. They estimated that the adjusted total mean annualized direct cost to payers was $27,259 per participant based on 624 established patients with NeP. Several previous studies (Gore et al., 2006; DiBonaventura et al., 2011) that investigated painful diabetic peripheral neuropathy found that a high severity of pain is associated with a high intake of medications, the increased use of healthcare resources, and the loss of productivity. This study supports the notion that medical costs for sufferers of chronic NeP are immense and impose a heavy burden on medical economics.

### 4.4 Limitations

Several NeP scales can be used to diagnose NeP, including painDETECT, the Douleur Neuropathique en Questions (DN4; Bouhassira, 2005), the Leeds Assessment of Neuropathic Symptoms and Signs (LANSS; Bennett, 2005) and the NeP Questionnaire (NPQ; Backonja, 2003). These questionnaires include approximately the same number of questions (DN4, 10 items; LANSS, 7 items; painDETECT and NPQ, 12 items each) regarding the perception of various types of pain. The DN4 might be more suitable for large population‐based surveys because of its relatively high sensitivity (93%) compared with that of the LANSS (36%) or the painDETECT (68%; Hallstrom and Norrbrink, 2011; Mathieson et al., 2015). We adopted the painDETECT to detect a neuropathic component because it is the only NeP questionnaire with a validated Japanese version to date. The painDETECT is also the only instrument that assesses the degree of perceived suffering. This enabled this study to find that the intensities of burning pain and pressure pain had the greatest influence on QOL, and in contrast, that the degree of numbness was only minimally associated with QOL. An awareness of perceived suffering helps identify pain characteristics and appropriately alert healthcare providers, even in situations in which the origin of the pain remains unclear or a pain specialist is not present. Thus, one limitation of our study is that we might not have used the most effective questionnaire.

Another limitation was the relatively low response rate (54.4%) to our survey, which might have affected the accuracy of our prevalence estimations. Bouhassira et al. (Bouhassira et al., 2008) conducted a postal survey to detect the prevalence rate of neuropathic pain with DN4 and showed a much higher response rate in France (83%). However, there were only 11 simple questions in their study to ensure maximal response rate. The questionnaire we used in this study had more than 50 questions related to not only neuropathic component but also pain characteristics, QOL and psychosocial factors that allowed us to recognize the burden of FBSS. Furthermore, the response rate in this study was still higher than that in other epidemiological studies of NeP [52% in France, (Torrance et al., 2006) and 42.5% in North America, (Toth et al., 2009)]”.

## 5 Conclusions

This population‐based epidemiological survey is the first to report the prevalence of chronic NeP in an Asian population. This study revealed that chronic NeP greatly affects physical and mental health as well as the social and daily lives of those with this condition. Recognizing the distress of chronic NeP sufferers might improve the quality of treatment for NeP and is essential for establishing national public health prevention strategies for NeP.

## Author contributions

S.I., T.U., T.T., M.N. and T.Y. designed this survey. Nippon Research Centre (Tokyo, Japan) and S.I. collected and analysed the data. S.I. drafted the manuscript under the supervision of T.U., T.T., M.N. and T.Y. All authors discussed the results and provided final approval of the version to be published.
